# Atmospheric ionization and cloud radiative forcing

**DOI:** 10.1038/s41598-021-99033-1

**Published:** 2021-10-11

**Authors:** Henrik Svensmark, Jacob Svensmark, Martin Bødker Enghoff, Nir J. Shaviv

**Affiliations:** 1 National Space Institute, Technical University of Denmark, Elektrovej 327, 2800 Kongens Lyngby, Denmark; 2 Niels Bohr Institute, University of Copenhagen, Niels Bohr Building, Jagtvej 128, 2. floor, 2200 Copenhagen N., Denmark; 3 Racah Institute of Physics, Hebrew University of Jerusalem, Giv’at Ram, Jerusalem, 91904 Israel

**Keywords:** Climate sciences, Astronomy and planetary science

## Abstract

Atmospheric ionization produced by cosmic rays has been suspected to influence aerosols and clouds, but its actual importance has been questioned. If changes in atmospheric ionization have a substantial impact on clouds, one would expect to observe significant responses in Earth’s energy budget. Here it is shown that the average of the five strongest week-long decreases in atmospheric ionization coincides with changes in the average net radiative balance of 1.7 W/m$^2$ (median value: 1.2 W/m$^2$) using CERES satellite observations. Simultaneous satellite observations of clouds show that these variations are mainly caused by changes in the short-wave radiation of low liquid clouds along with small changes in the long-wave radiation, and are almost exclusively located over the pristine areas of the oceans. These observed radiation and cloud changes are consistent with a link in which atmospheric ionization modulates aerosol's formation and growth, which survive to cloud condensation nuclei and ultimately affect cloud formation and thereby temporarily the radiative balance of Earth.

## Introduction

A fundamental question during the last two decades has been whether changes in atmospheric ionization can perturb aerosols and thereby cloud properties. Both aerosols and clouds are an essential part of the terrestrial atmosphere, influencing weather and climate^1–3^. Ionization in the atmosphere is mainly caused by cosmic ray particles, which have their origin outside our solar system. Solar activity modulates the flux of cosmic ray particles on time scales from days to millennia, whereas on geological time scales it is the position of the solar system in our Galaxy, which is important for ionization^4,5^. A link involving ionization, aerosols, and clouds would be an exciting interconnection between Earth and the Galaxy. Indeed, laboratory experiments demonstrated that ions assist the nucleation of new ($\sim$ 1–2 nm) aerosol particles^6,7^, and evidence from airborne observations also document that ion nucleation is an important source of aerosols in the free troposphere^8^. However, these results do not by themselves assure that aerosols survive to cloud condensation nuclei (CCN) such that variations in ionization can change CCN concentrations and subsequently clouds. In fact, studies testing the role of ion-nucleation using global numerical aerosol models indicate that the response of CCN to changes in ionization is too small to be of any significance^9–14^. The reason being that any changes in ion-nucleated aerosol number density get attenuated by absorption on existing aerosols before the particles can grow to CCN. However, there is evidence contrasting the numerical results. Recent theoretical and experimental results detail how ions can accelerate the growth of small aerosols by increasing the mass-flux from the gas phase to aerosols^15,16^, and this ion-condensation mechanism is not included in the numerical modeling. A mechanism accelerating aerosol growth in the real atmosphere would lead to a higher survival rate of aerosols growing to CCN sizes.

Observational support for the link connecting atmospheric ionization with cloud changes has been pursued using naturally occurring week-long suppression of atmospheric ionization of the order 10–20%. Such events are called Forbush Decreases (FDs)^17^ and are caused by a magnetized plasma cloud from the Sun hitting Earth, thereby shielding part of the cosmic ray flux. Initially, FD studies gave conflicting results^18–23^ (see section 7.4 in^23^) but by sorting the FDs according to their strength a significant response was found in both aerosols and clouds in the case of the strong FDs^23^.

However, for the ion-aerosol-cloud link to be important, the changes in atmospheric ionization should significantly influence Earth’s energy balance. Here we address this question by using satellite data from Clouds and the Earth’s Radiant System (CERES)^24^ of top-of-atmosphere (TOA CERES daily SYN1deg) radiative forcing during strong FDs together with simultaneous cloud observation from the Moderate Resolution Imaging Spectroradiometer (MODIS)^25^ (See Methods). The thirteen strongest FDs after 2000, where the CERES and MODIS instruments have been active, have been ranked according to their ionization strength in the atmosphere (see Table 1). It is shown that a $\sim$10% decrease in cosmic ray ionization of the five strongest FDs results in a global top-of-atmosphere radiative forcing of 1–2 W/m$^2$ with a delay of $\sim$ 5–7 days caused by mainly low liquid clouds over the oceans. Deep convective clouds also react to the FD, but here the net effect on the radiative budget is muted due to opposing effects of short-wave and long-wave forcing.

**Figure 1:**
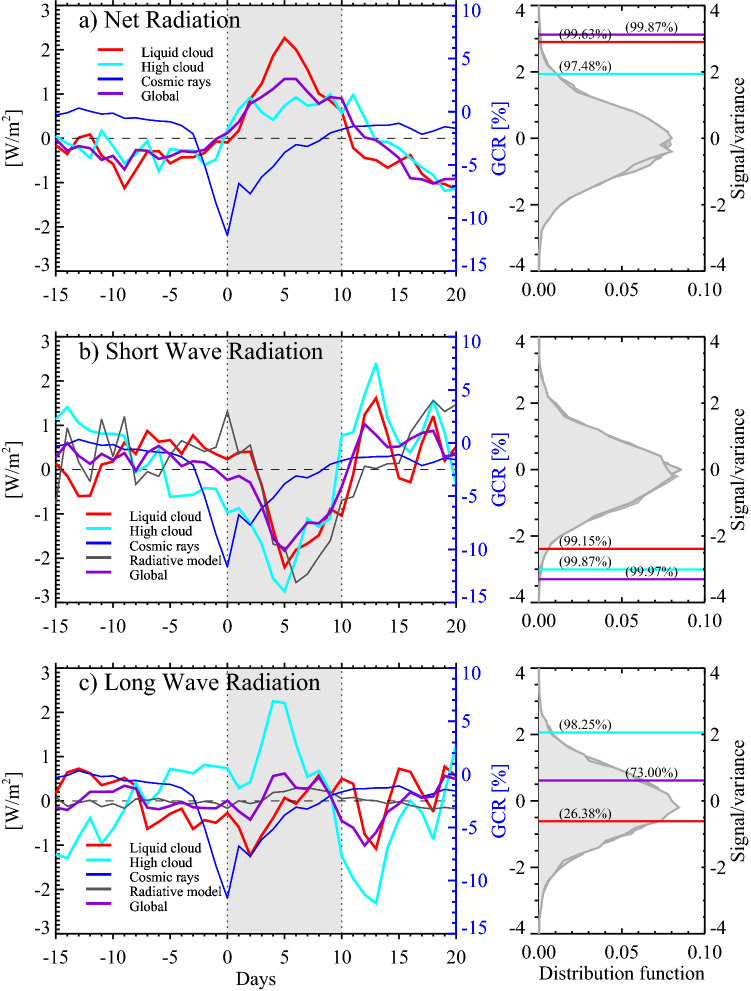
The top of atmosphere radiative balance (relative to the mean of the time series, left column) from CERES data superposed for the five strongest FDs since 2000. The panel **(a)** is the change in net radiative forcing for three situations: geographical areas with predominantly, (1) liquid clouds (red curve), (2) areas with high clouds (cyan curve), (3) and global averages (purple curve). The gray area signifies the period used to integrate the response (day 0–10). The blue curve is the variation in cosmic rays as measured by the Oulu neutron monitor. Right-hand columns are bootstrap distribution functions of the integrated signal. The red, cyan, and purple lines denote the significance of the FD signal obtained from the three distribution functions. Panel **(b)** left-hand side shows changes in TOA short wave radiation for areas with liquid clouds (red curve), high clouds (cyan curve), and globally (purple curve). Results of the Fu-Liou radiative transfer model of changes in TOA SW radiative forcing obtained using the observed changes in MODIS cloud parameters are described by the gray curve. The right-hand panel **(b)** shows the achieved significance of the FD signals. Finally, panel **(c)** is the change in TOA LW radiative forcing similarly as for SW in panel **(b)**.

## Results

One of the main problems in demonstrating significant responses in aerosols and clouds to sudden changes in ionization during a FD event is the dominant meteorological noise that tends to mask any signal. Another problem is large differences in the resulting decrease in ionization between individual FD events. A possible solution to this problem is to rank the FDs according to their strength and use the strongest FD events to search for significant signals. This strategy has been used previously, where in addition to using the strength, the response was integrated over 10 days following the FD minimum in cosmic rays^23^. This procedure takes possible auto-correlation into account, i.e., the situation where the signal is distributed over a few days. The significance of the obtained signal can then be evaluated relative to a Monte-Carlo simulation using random FD dates (details in Methods). This approach will now be used on the CERES data.

**Figure 2:**
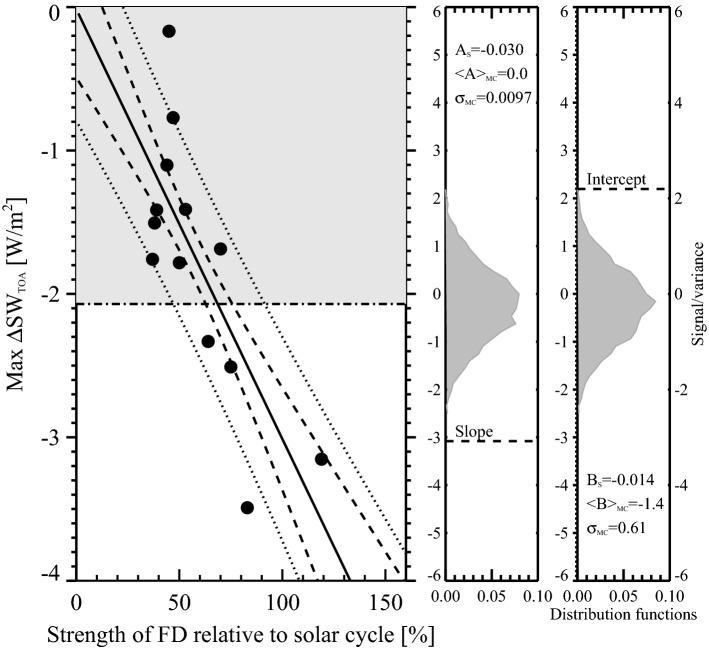
The response of CERES short-wave radiation as a function of FD strength. The left panel shows the most substantial deviation between day 0 and day 10 in short wave radiation globaly averaged for each Forbush decrease as a function of the FD strength (dots). The black line is a linear fit to the 13 data points and the dashed lines and dotted lines represent two and four standard deviations of the fit, respectively. The dash-dotted line is the mean of the most substantial deviation between day 0 and day 10 using 10$^4$ Monte Carlo simulations with random FD dates. The black circles are the 13 FDs. The two panels to the right display the distribution functions of the slope and intercept of the linear fit, based on 10$^4$ Monte Carlo simulations using CERES SW data with random dates for the FD. Note that the significance of the above linear slope is $\sim$3$\sigma$, and that the Monte Carlo simulations using random dates for the FD events result in an average zero slope as expected.

### Globally averaged responses

For a given FD event we consider a period beginning 15 days before the FD minimum in the galactic cosmic ray (GCR) flux to 20 days after. After global averaging, all 36-day time series have a linear trend removed (see Methods) to account for seasonal effects. The left column of Fig. 1 shows the responses in (a) NET radiation, (b) SW radiation, and (c) LW radiation in the CERES instrument at TOA superposed for the five strongest FD events. Only the five strongest events are used as adding more events decreases the signal-to-noise ratio due to the weaker strength of the rest of the events (see Methods). The right column shows the distribution function and the achieved significance level of the integrated signals (gray area from day 0 to 10 in the left panels of Fig. 1) based on 10$^4$ Monte Carlo bootstrap samples of the integrated/summed response between day 0 to 10 using random FD dates in the full CERES time series 2000–2006^23^ (see Methods). Figure 1a shows the NET radiation for three relevant situations: Areas with liquid cloud fraction > 0.25 (red curve), areas with high cloud fraction > 0.25 (cyan curve), and globally (purple curve). The areas of high or liquid clouds are defined as an average cloud fraction of more than 0.25 in the corresponding MODIS parameter averaged over the period 2000-2006. The fractional areas of Earth with a given cloud type above the threshold level are: Area (Liquid clouds) = 0.52, area (High clouds) = 0.44, and area (Ice clouds) = 0.40. Note in Fig. 1a that approximately 5 days after the GCR minimum (blue curve) an increased flux of $\sim$1–2 W/m$^2$ is entering the Earth system.

**Figure 3:**
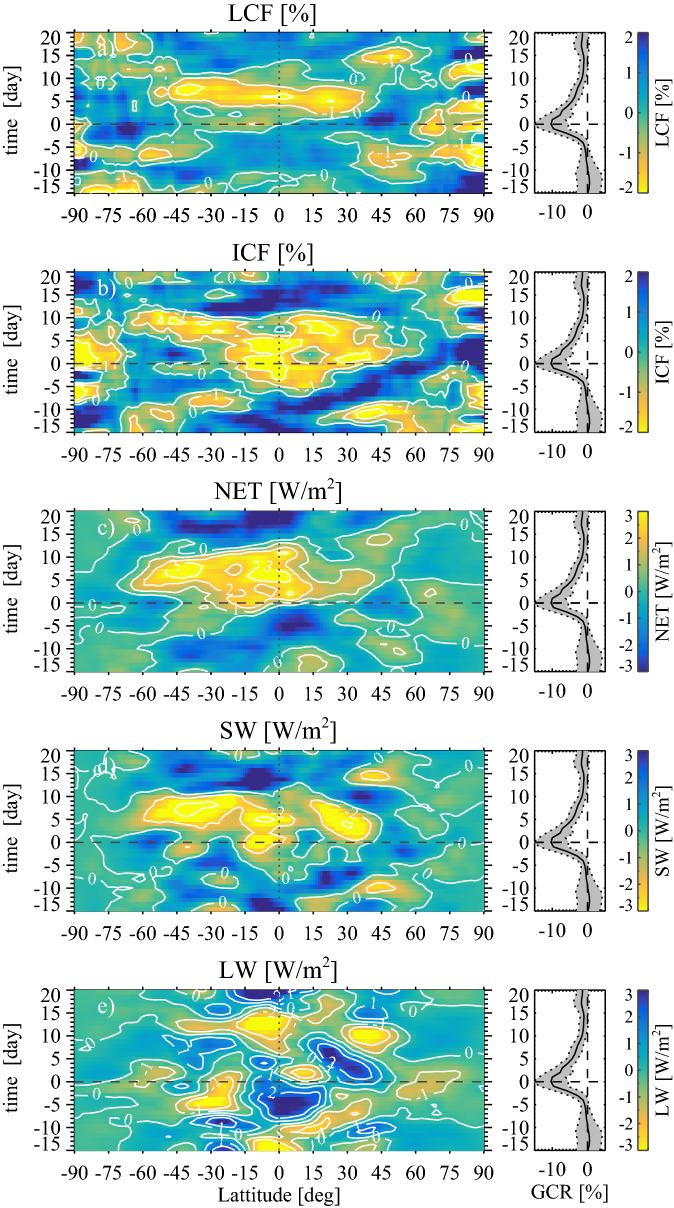
Maps depicting changes in clouds and TOA radiative balance as a function of latitude and time (centered on the FD minimum). The maps are superposed data from the five strongest FDs after 2000. **(a)** The left panel is based on liquid cloud fraction observations from MODIS in (%). The right panel in all rows displays the change in cosmic rays during the same period (black curve). **(b)** Left panel is ice-cloud-fraction observations from MODIS in (%). **(c)** Left panel is TOA CERES NET radiation in (W/m$^2$). **(d)** Left panel is TOA CERES SW radiation in (W/m$^2$). **(e)** Shows the LW similar to the SW in **(d)**.

Figure 1b displays the SW radiation at the TOA, again separated into areas with predominantly liquid clouds, areas with mostly high clouds, and globally. Here a minimum about five days after the GCR minimum is seen where $\sim$2–3 W/m$^2$ less SW radiation is reflected from Earth. The Fu-Liou radiative transfer model^26,27^ is used to compare the change in SW and LW at TOA using built-in response functions for the CERES SW and LW instruments. The inputs to the model are simultaneous MODIS observations of liquid cloud changes of cloud fraction, optical thickness, and effective droplet radius, superposed over the same 5 FDs (grey curve), using a low cloud height between 900 and 700 hPa. The right-hand panel of Fig. 1b depicts the achieved significance levels. Finally, Fig. 1c is similar to Fig. 1b but for the change in LW. The gray curve is the LW result of the Fu-Liou model. See Methods for how TOA Net, SW, and LW are measured.

Figure 2 illustrates the relevance of using the FD strength by superposing data for the five strongest FD as is done in the above Fig. 1. In Fig. 2 the largest deviation in global SW radiation between day 0 and day 10 is plotted as a function of the strength of the individual 13 FDs from Table 1, with a linear fit to the data. Using all available CERES data with random FD dates it is possible to estimate the mean of the largest deviation over a 10 day period and compare this value with the actual FD dates. The mean of the largest deviations is shown as the horizontal dash-dotted line in Fig. 2. It is seen that black points with the lowest strength ($<60$%) are mainly below this value and therefore in the noise. The group of black points with strength larger than 60% are mainly above this level. The right-hand panels of Fig. 2 are the distribution functions of the slope and intercept of linear fits using all 13 random FD dates based on 10$^4$ Monte Carlo simulation. Note that the slope and intercept of the real FD data have a significance corresponding to three $\sigma$, demonstrating a large statistical signicance between FD strength and the response as defined above.

Although the linear trend has a high significance, it does not exclude the possibility that a single strong event dominates the overall signal. As can be seen in Fig. 2 FD 2, which occurred January 19, 2003, has the largest response of all the FDs. Consequently, statistical tests were performed to see if FD 2 is an outlier. Although the definition of outliers is a quarrelsome issue, there are statistical methods to test if a data point classifies as an outlier^28^. First, the deviation, $\Delta y$, of the FD response from the linear regression line seen in Fig. 2, was calculated. The 13 $\Delta y$ data points underwent several tests: Z-score, Pierce test, Grubbs’s test, and Dixon’s Q-test. They all predicted that FD2 is not an outlier and it is therefore kept in the sample.

As can be seen in Fig. 2 the weak FDs have comparatively weak responses. It is therefore important to optimize the signal to noise ratio. Due to the varying FD strength (see Methods), the signal-to noise-ratio has a local maximum when superposing the five strongest FDs. The maximum in signal-to-noise ratio is the argument for using the superposition of the five strongest FDs to demonstrate significance of FD signals in CERES and MODIS.

**Figure 4:**
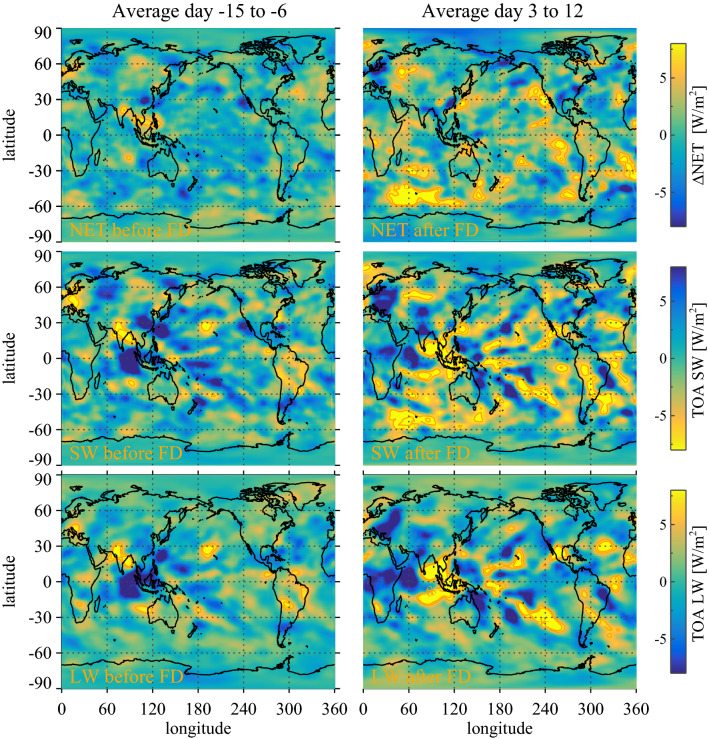
The left-hand column represents variations in the average radiative state before the FD (for details see Methods) averaged over the first nine days before the FD minimum for the NET, SW, and LW, respectively, and the right-hand column is the variations in the average radiative state after the FD averaged over nine days after the FD starting on day 3. Top panels are NET radiation. Middle panels are for SW radiation, and finally, the bottom panel is LW radiation.

**Figure 5:**
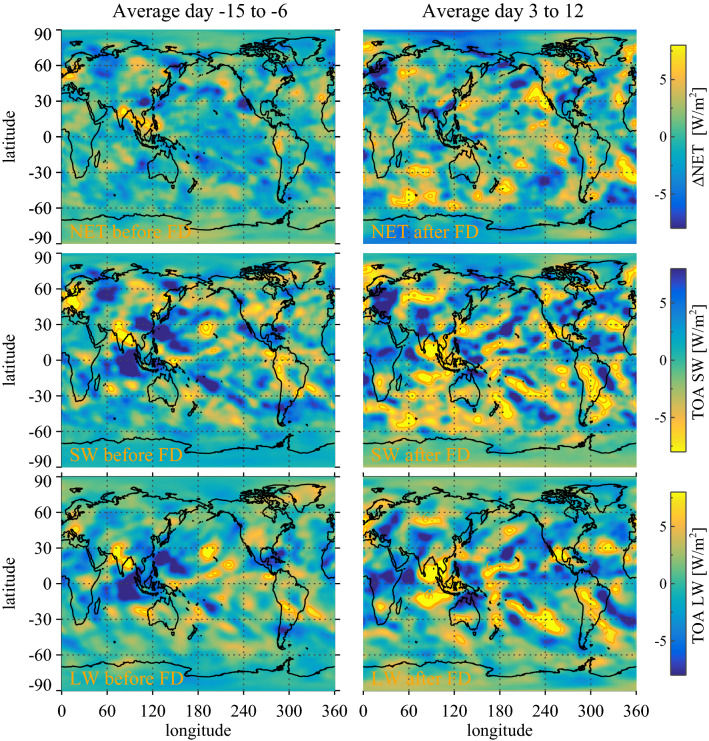
Same figure as Fig. 4 but without the strong FD2 event. Note that the overall patterns are similar to Fig. 4 but as expected a little weaker.

### Zonal responses

Figure 3 illustrates the zonal (latitudes $-90^{\circ }$ $- 90^{\circ }$) and temporal changes (36-day time series) in clouds together with simultaneous changes in the global TOA radiative balance. The zonal maps in the left column are again the average of the five strongest FDs. The column on the right-hand side shows the relative change in GCR (black curve), and the 1-$\sigma$ scatter (gray band) using a box-car average of 3 days, measured in (%) relative to the base level between day −15 and −5. Figure 3a is the change in MODIS liquid cloud fraction in (%). Figure 3b are ice-cloud-fraction observations from MODIS in (%). Figure 3c shows the TOA CERES NET radiation in (W/m$^2$). Note the response approximately 5 days after the FD minimum in the latitude range −50$^{\circ }$ to 40$^{\circ }$. Figure 3d shows TOA CERES SW radiation in (W/m$^2$), and finally Fig. 3e is the change in TOA LW in (W/m$^2$).

### Global maps

It is possible to obtain a global map of changes in the radiative balance averaged over the five strongest FDs. Figure 4’s top panels show on the left-hand side a global map of the NET radiation before the FD-minimum, averaged over the period day −15 to day −6 before the minimum in FD ionization. It represents variations in the average NET radiative state before the FD (for details see “Methods”). The global map on the right-hand side is the NET radiation change after the FD-minimum averaged over day 3 to day 12 to accentuate the response. Similarly, the middle panel graphs the SW response of Fig. 4, while the bottom panel shows the LW response of Fig. 4. Although FD2 is the strongest, by excluding it from the map in Fig. 4 we show that the remaining four strongest FD still produce quite a similar pattern of responses, as can be seen from Fig. 5. Note that the Monte Carlo statistics used in the globally averaged signals, as in Fig. 1, are one way of testing the significance of the maps in Fig. 4, i.e. corresponding to the summation of the above maps.

**Figure 6:**
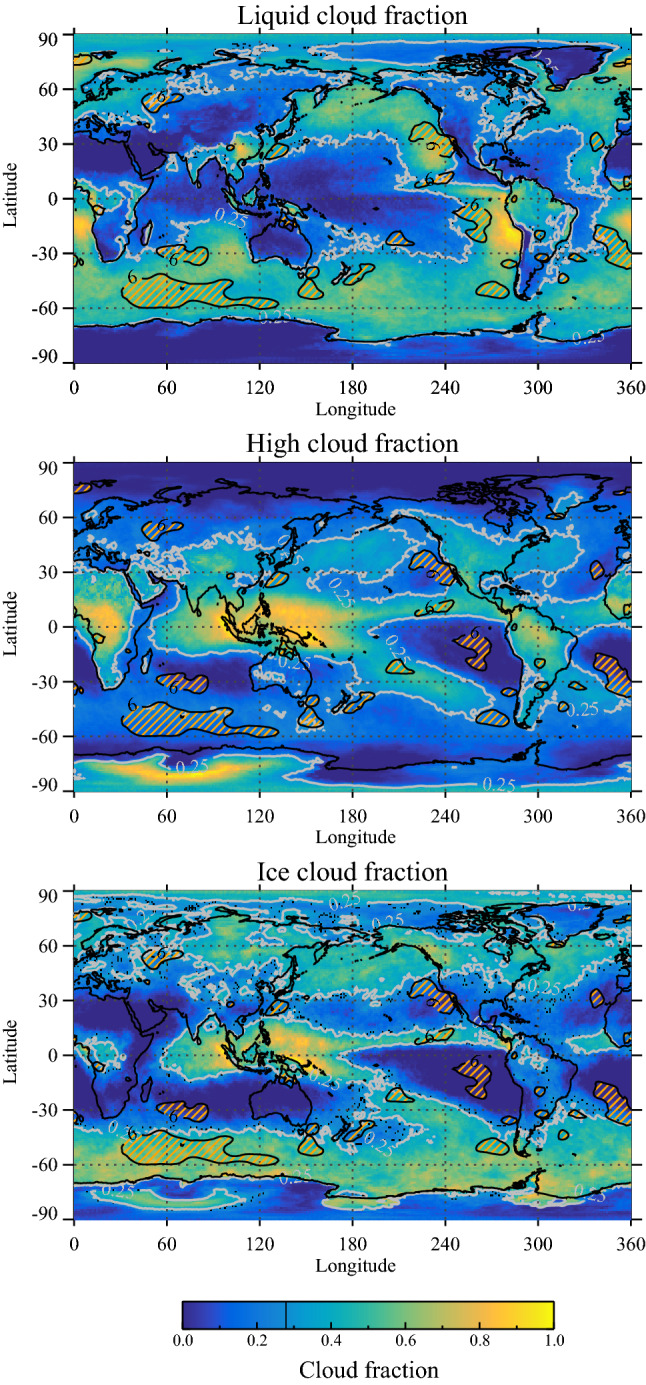
Global maps of three cloud types based on MODIS observations. The top panel is regions with liquid clouds (mainly the green regions). The middle panel is high clouds. The bottom panel is the ice-cloud-fraction. The gray contour lines on all three maps show threshold cloud fraction 0.25 used for the cloud masks. The fractional area of Earth with cloud types exceeding the threshold are: a (Liquid clouds) = 0.52, a (High clouds) = 0.44 and, a (Ice clouds) = 0.40. Superposed on all three maps are the (orange hatched) regions with large responses found in CERES TOA net radiation following the minimum in cosmic rays (black contour lines are 6 W/m$^2$). Note that these regions are almost exclusively confined to areas dominated by liquid clouds.

Figure 6 displays three panels of Earth with liquid, high, and ice cloud frequency. Superposed on all three sub-figures are the regions with the largest responses in NET radiation (see Fig. 4 top right panel). The top panel demonstrates that the strongest signal in NET radiation is almost exclusively regions with low clouds.

## Discussion

The present results combine statistical correlations with expectations from atmospheric physics. If a link between ionization, aerosols, and clouds exists, it is reasonable to expect that the strength of an FD should influence the size of an atmospheric response in clouds and radiative forcing. Figure 2 gives a demonstration of a linear relationship between the strength of 13 FD and the global SW responses. Testing the significance by a Monte Carlo simulation using random dates showed that the slope of the linear relationship was significant at the three $\sigma$ level. As can be observed from Fig. 2 FD2 has the strongest response, and it could be that the event is so dominant that it defines the response. Statistical tests did not classify the FD2 SW response as an outlier relative to the other 12 FDs and was therefore kept in the sample.

Figure 1 offers insight into the response of the terrestrial cloud system during strong FDs. The global signals (purple curves) of the TOA NET, and SW radiation in Fig. 1 show a significant response, reaching a 2–3 W/m$^2$ perturbation around 5 days after the FD minimum. An increase is seen in the NET radiation, while the SW exhibits a complementary decrease. Notably, the LW component in the bottom panel shows no response to the FDs. By applying cloud masks selecting for either liquid cloud or for high clouds, it is possible to understand the response.

Considering first the liquid clouds (red curves), the signals in the NET and SW radiation in Fig. 1 persist and even strengthen, while the LW component remains almost unaffected. The explanation is that liquid clouds are low clouds, and therefore the difference between cloud top temperature and Earth’s surface temperature is relatively small. Thus, a decrease in liquid cloud fraction would manifest as a nearly unchanged LW signal. In contrast, liquid clouds affect SW radiation, which increases NET-absorbed radiation. This scenario is corroborated by using the Fu-Liou radiative transfer model^26,27^ using input of simultaneous measured MODIS liquid cloud parameters ( cloud fraction, cloud optical thickness, and effective cloud droplet radius) as displayed in Fig. 1b for SW changes (gray curve) and Fig. 1c for LW (gray curve).

Applying a cloud mask to extract high cloud regions leads to a different result (Fig. 1, cyan curves). Here, the response in NET radiation is relatively smaller and less significant. However, the effects in the SW and LW radiation are both significant and closely mirror each other, which is consistent with expectations from a decrease in the high cloud fraction. Fewer high clouds will lead to less SW radiation reflected out to space (negative SW at TOA). However, since a large fraction of the high clouds are deep convective clouds, there will be a large LW contrast between the cold cloud top and the warm terrestrial surface. A decrease in high clouds will, therefore, increase the LW radiation escaping to space. As a consequence^29^, the change in SW and LW will approximately cancel each other resulting in the smaller NET radiation signal $\sim$1 W/m$^2$, as seen in Fig. 1.

The connection between CERES TOA radiation balance and clouds can be further consolidated when studying the zonal temporal responses, as shown in Fig. 3. Figure 3a shows the response in MODIS liquid cloud fraction with minimum 5–7 days after day 0 with a longer response time seemingly in the southern hemisphere. The zonal distribution of the response is almost symmetric around the equator ranging from −50$^\circ$ to 40$^\circ$, but suggestive of a stronger response in the southern hemisphere. The average solar zenith angle for the 5 FD events was −0.6$^\circ$, which does not in itself indicate a solar angle preference for either hemisphere. Figure 3b expresses the response in ice clouds. The main difference between ice clouds and the liquid clouds is that the temporal response close to the equator starts earlier in the ice clouds. This difference may be due to the much larger vertical velocities in the deep convective clouds, which activate aerosols at even smaller sizes than in the low liquid clouds due to a higher supersaturation of water vapor. Alternatively, the time difference could be due to nucleation happening high in the troposphere followed by aerosol growth as they descend^30^ making them available as CCN later at the heights were liquid cloud formation takes place.

NET and SW response in Fig. 3c,d, have similarities with maximum changes of 3 and −4 W/m$^2$, respectively, and both have a well defined maximum/minimum around day 5. A response extremum at day 5 in the clouds and NET radiation as seen in Fig. 3a,c indicate that the observed effect can not be a result of meridional transport since typical meridional wind speeds are of the order of 1 m/s. Therefore an ion-effect on the time scales of days has to be relatively confined to a particular latitude.

The effect of FDs on the overall radiative balance is visible from the global maps shown in Fig. 4. Figure 4 illustrates the radiative responses in NET, SW, and LW (visible in the right-hand panels). Comparing these responses with the cloud distributions of liquid clouds, high clouds, and ice clouds of Fig. 6 provide valuable information on the workings of the ion-cloud link. From Fig. 4 (top right panel) one see the response in NET radiation and comparing the maximum responses with the distribution of liquid clouds Fig. 6 (top panel) the NET responses are mainly over the clean southern oceans and where low clouds dominate. Furthermore, comparing Fig. 6 (middle right panel) with Fig. 4 LW (bottom right panels) it is seen that large responses in LW are mainly confined to regions with a high frequency of deep convective clouds. The same LW regions can be seen in the SW map in Fig. 4 (middle panels) with a near cancellation of LW and SW signals, in agreement with the well-known effect of deep convective clouds having significant but compensating effects in the LW and SW that largely cancel^29^. These observations therefore imply that the main NET effect of FDs on the radiative budget is from low liquid clouds caused by the change in the cloud fraction with SW albedo changes dominating. Although FD 2 is the strongest, by excluding it from the map in Fig. 4 we demonstrate that the remaining four strongest FD still produced quite a similar pattern of responses, as can be seen from Fig. 5.

A further complication to FD 2 is that one of the strongest ground level events occurred within the FD decrease, which has the opposite effect of the FD by increasing the ionization. The acceleration of particles in a ground-level event is due to solar activity. The particle energy is rarely more than 10 GeV (much lower than Galactic cosmic rays) and with a peak lasting less than an hour. The solar accelerated particles are therefore usually too weak to penetrate Earth's magnetic field at low latitudes. Neutron monitor counts of daily-mean show that the ground level event does not dominate the FD2 event for latitudes below approximately 55 deg., corresponding to more than 80% of Earth. However, the ground-level event dominates in the polar regions, with a significant excess of atmospheric ionization. From the cosmic ray, aerosol, and cloud link, one would expect a decrease in cosmic rays, aerosol, and cloud over most of Earth, except in polar regions, where the increased ionization should produce an excess of aerosols. Such an increase has been seen^31^. Consequently, this unique feature of FD2 provides an additional independent consistency test of the cosmic-ray-aerosol link.

In summary, the results presented show that

- A statistically significant linear relationship exists between the strength of the 13 FDs and the globally averaged SW responses.The presented results are mainly based on an average of the five strongest Forbush events, which ensured that the signal-to-noise ratio was a maximum. By only having five strong Forbush decreases, there is a limit to the robustness of the presented results. Nonetheless, removing Forbush event 2 from the calculation of the global net radiation change still produces a significant result of 1.2 W/m$^2$ compared to 1.7 W/m$^2$ with FD2 included.
- Following the minimum in cosmic rays, one observes a delay of 5-7 days in clouds and radiative response as expected from the time it takes aerosols to grow from nucleated sizes (1–2 nm) to cloud condensation nuclei. Clouds in the tropics which contain ice are mainly deep convective clouds. Here the updraft velocity is much larger and, therefore, also the supersaturation^32^. Higher supersaturation activates much smaller sizes of aerosols as CCN. As a consequence, a shorter delay is expected in the response, as seen.
- The second strongest FD 2 is exceptional by the occurrence of a ground-level event within the period of the FD 2. The consequence is a decrease in ionization in more than 80% of the area of Earth and an increase in polar regions. Observation in polar regions and the rest of the Earth demonstrates a decrease of aerosols^21,23^ and an increase in polar regions^31^. These and the present observations are consistent with a cosmic ray-aerosol-cloud link.
- The zonal responses in liquid clouds show a delay in minimum SW between 50° to 45° with a slight tendency towards a longer delay in the southern hemisphere (see Fig. 3a,d). A longer delay in the southern hemisphere is consistent with a lower trace-gas concentration (from which particles grow) due to a more clean atmosphere.
- From the spatial maps, it is clear that the primary responses are over the oceans and that low liquid clouds are mainly responsible for the change in net radiative forcing. The global response in net radiation to the average of the five strongest FD is approximately 2 W/m$^2$.

A consistent picture is emerging, suggesting that variations in ionization are connected to aerosols and clouds^21,23^, and now also the energy budget. In fact, the aerosol–cloud observations get a simple explanation by the ion–aerosol–cloud hypothesis. A decrease in atmospheric ionization decreases the number of ion-nucleated aerosols (1–1.5 nm). This generation of fewer small aerosols then has to grow to CCN sizes ($\sim$50 nm) where they can influence clouds. In the free troposphere it typically takes $\sim$5 days (depending on the supersaturation) for aerosols to grow to CCN^33^, which is consistent with the observed delay seen in Figs. 1 and 3. One implication of a cosmic ray-aerosol-cloud link is that numerical models are missing relevant physics related to ion and aerosol interactions. Specifically, it was shown that the electrostatic interactions between ions and aerosols continuously add mass to the aerosols at a rate depending on the ion-concentration^16^. Therefore, by changing the ion concentration the aerosol growth rate will also change, which is important for the survival of aerosols to CCN. This ion-aerosol growth acceleration mechanism was found to be important under the following conditions: (1) aerosols are smaller than $\sim$25 nm, (2) the total number of aerosol particles is small, and (3) the condensing gas concentration (e.g. H$_2$SO$_4$) is low^16,34^. The first condition is important for aerosol survival and the last two conditions are typically fulfilled over the remote oceans^8^. For example, low CCN concentrations result in low droplet concentrations in marine stratus clouds (N < 100 cm$^{-3}$) as can be found over large parts of the remote oceans^35–37^. Ion–aerosol interactions and aerosol survival to CCN is therefore suggested to be sensitive to the prevailing ion densities and are capable of modifying clouds and the radiative energy budget in the secluded atmosphere over the oceans.

## Methods

### Data

The data employed in this work originate from three sources: (1) Daily GCR counts are obtained from the Oulu neutron monitor (http://cosmicrays.oulu.fi/). (2) The ordered Forbush decrease (FD) list is obtained from Ref.^23^, and listed in Table 1 for reference. (3) Data on Earth’s radiative balance is obtained from the NASA satellite program, “Clouds and the Earth’s Radiant Energy System” (CERES)^24^. The data are “Observed Radiative Fluxes” (SYN1deg-Day) product of daily averages (https://eosweb.larc.nasa.gov/project/ceres/syn1deg-day_ed3a_table). The specific parameters used are, top-of-atmosphere (TOA) outgoing (reflected) short wave radiation between 0.3$\,\upmu$m and 5.0$\upmu$m (SW), this observed broadband shortwave reflected (upwelling) flux at the top of the atmosphere (TOA—around 20 km altitude) is defined as positive. TOA outgoing longwave between 5.0$\,\upmu$m and 125$\,\upmu$m (LW) is the (upwelling) thermal outgoing LW flux at the top of the atmosphere and is defined as positive. The Net TOA Flux, is the broadband incoming solar (downwelling) minus the reflected shortwave (SW) (upwelling) and longwave (LW) emitted (up-welling) flux at the top of the atmosphere. If the Net Flux is positive the Earth is warming, while when negative the Earth is cooling. (4) Cloud data are obtained from the Moderate Resolution Imaging Spectroradiometer (MODIS)^25^. Daily averages of the following key cloud parameters are extracted: (1) Cloud effective emissivity, (2) Cloud optical thickness, (3) Liquid water path (LWP), (4) Liquid water cloud fraction, (5) Liquid cloud effective radius ($R_{\rm {eff}}$), (6) Column density of CCNs, (7) Ice-cloud-fraction, (8) High cloud inferred fraction. All data from the “MOD08_D3” product.

**Table 1:** The 13 most influential FD events since 2000 during which the CERES and MODIS instruments have been active, sorted by strength.

Order	Date	Decrease (%)
1	31/10/2003	119
2	19/1/2005	83
3	13/9/2005	75
4	16/7/2000	70
5	12/4/2001	64
6	10/11/2004	53
7	26/9/2001	50
8	17/7/2005	47
9	27/7/2004	45
10	31/5/2003	44
11	25/11/2001	39
12	15/5/2005	38
13	28/8/2001	37

The first and second columns are, respectively, the order and date of minimum cosmic ray flux. The third column shows the percentage decrease in the ion production *relative* to the reduction in cosmic rays from solar minimum to solar maximum over a solar cycle, i.e., FD 1 changed the GCR flux more than the average change over the solar cycle^23^.Note that while there have been several FDs since the latest on the list (Sept. 13th 2005) none of them have been strong enough to enter the top 5 of the list.

### Statistics

A Monte Carlo bootstrap method determines the statistical significance of the globally averaged signals. CERES data of SW, LW and NET radiation are in the form of global maps, $U(i,j,t_n)$ where index $i \in [1;180]$, $j \in [1;360]$ (1$\times$1$^\circ$ resolution) and a temporal resolution of 1 day, covering the period 1st March 2000 to 31st December 2005. The same holds for the MODIS cloud parameters. Global spatial averages over 36 days consisting of 15 days before the FD minimum (on day 0) and 20 days after are given by

1 $$\begin{aligned} \langle U(t_n)\rangle = \sum _{j=1}^{360} \sum _{i=-90}^{90} U(i,j,t_n) \cos [\phi (i)] ~/~ \sum _{i=-90}^{90} \cos [\phi (i)], \;\; t_n \in [-15,20], \end{aligned}$$

where $\cos [\phi (i)]$ is the area correction as a function of latitude $\phi (i)$. The zonal averages are functions of both time and latitude:

2 $$\begin{aligned} \langle U(i,t_n)\rangle = \sum _{j=1}^{360} U(i,j,t_n) /360. \end{aligned}$$

A linear trend is removed from the temporal variability in all data. For global averages, each 36-day time series is de-trended:

3 $$\begin{aligned} \langle U(t_n)\rangle _0 = \langle U(t_n)\rangle - a t_n - b, \end{aligned}$$

where *a*, *b* are best linear fit coefficients. For a zonal averages, we de-trend temporally on a latitude basis, such that

4 $$\begin{aligned} \langle U(i,t_n)\rangle _0 = \langle U(i,t_n)\rangle - a_i t_n - b_i, \end{aligned}$$

where $a_i,b_i$ are the best linear fit coefficients for the time-series at latitude *i*. Finally for global maps we de-trend on a pixel-by-pixel level, such that

5 $$\begin{aligned} \langle U(i,j,t_n)\rangle _0 = \langle U(i,j,t_n)\rangle - a_{i,j} t_n - b_{i,j}, \end{aligned}$$

where $a_{i,j},b_{i,j}$ are the best linear fit coefficients for the time-series at latitude *i* and longitude *j*.

The above 36 days time series for global averages, zonal means and maps, abbreviated as $U_k$ for the *k*’th strongest FD, may then be averaged over the five strongest events:

6 $$\begin{aligned} \langle U\rangle _{FD} = \frac{1}{5}\sum _{k=1}^{5} \langle U_{k}\rangle _0 . \end{aligned}$$

Here *k* refers to each of the individual five strongest FD in Table 1.

Next, a statistic for the global response signal in an analysed satellite parameters can be defined. The time series in Eq. 6 for the global mean $\langle U(t_n)\rangle _{FD}$ can be integrated over the dates $t_n\in [t_1,t_2]$ where a signal is physically expected:

7 $$\begin{aligned} FS = \sum _{n=t_1}^{t_2}\langle U(t_n)\rangle _{FD}. \end{aligned}$$

For the present analysis we take $t_1=0$ and $t_2=10$. Then a Monte Carlo bootstrap statistic from similar constructs however with the GCR mininum date of the FD replaced with a random date within the full time series can be made. $N_B$ sets of 5 de-trended time series $\langle B_{i,j}(t_n)\rangle _0$ can be constructed in the same way as the FD time series as

8 $$\begin{aligned} \langle B_{j}(t_n)\rangle _0 = \frac{1}{5}\sum _{i=1}^{5} \langle B_{i,j}(t_n)\rangle _0, \end{aligned}$$

and their signal can be integrated (summed)

9 $$\begin{aligned} BS _j = \sum _{t=t_1}^{t_2} \langle B_{j}(t_n)\rangle _0. \end{aligned}$$

Here *j* is the bootstrap sample number. We want to know if

10 $$\begin{aligned} DFS = FS-\frac{1}{N_B}\sum _{j=1}^{N_B}BS_j \end{aligned}$$

is drawn from the same distribution as the bootstrap samples

11 $$\begin{aligned} DBS_j = BS_j - \frac{1}{N_B}\sum _{j=1}^{N_B}BS_j. \end{aligned}$$

Then, we define the statistic of the achieved significance level (26) as

12 $$\begin{aligned} ASL = \left[ \mathrm {Number\, of\, bootstrap\, samples\, where\,} DBS_j \ge DFS \right] / N_B. \end{aligned}$$

#### Statistical effect of having varying signal strength

If the *n* FD signals all have the same strength $S_1$ then the signal to noise ratio would grow as

13 $$\begin{aligned} \frac{S_n}{\sigma } = \sqrt{n} \;\; \frac{S_1}{\sigma _1} \end{aligned}$$

where $\sigma$ is the variance of the added noise, and $\sigma _1$ the noise of a single time series. This is the standard result if the signals all have the same strength. However, if the signals are of decreasing relative strength $f_n$, ($f_1$ = 1), then

14 $$\begin{aligned} \frac{S_n}{\sigma } = \sum _{i=1}^n \sqrt{n} \;\; \frac{f_i S_1}{\sigma _1} \end{aligned}$$

This function will in general not grow as $\sqrt{n}$ as in the general case, but can decrease and even have a local minimum. In the case of TOA SW data the signal to noise ratio has a local maximum at $n=5$ and decreases when more FD are added. This help the justification of using the five strongest FD in the present work.

Estimates of the strength of the individual FDs is detailed in^23^. The result was a list of 26 FD in the period 1983–2007, all ranked according to strength. There the change in cosmic rays primary spectrum at 1 AU was calculated using available neutron monitors and muon detectors from Nagoya. From this variation, one can estimate the change in atmospheric ionization using CORSIKA—COsmic Ray SImulations for KAscade, a Monte Carlo code to simulate extensive air showers. Table 1 show the ranking and timing of 13 FD events after 2000. Interestingly, there has been no strong FD since 2006. The first column is the date of the minimum in neutron counts during the FD, and the second column is the strength relative to the change in cosmic rays over a solar cycle. The FD event in 2003 had a larger drop in cosmic rays than the reference solar cycle modulation and is why the strength is set to 119%^23^.
